# A New Coordinate System for Magnetic Resonance Imaging of the Vestibular System

**DOI:** 10.3389/fneur.2021.789887

**Published:** 2022-01-05

**Authors:** Weixing Liu, Gui Chen, Junyang Xie, Tianhao Liang, Chunyi Zhang, Xiao Liao, Wenjing Liao, Lijuan Song, Xiaowen Zhang

**Affiliations:** State Key Laboratory of Respiratory Disease, Department of Otolaryngology–Head and Neck Surgery, The First Affiliated Hospital of Guangzhou Medical University, Guangzhou, China

**Keywords:** magnetic resonance imaging, coordinate system, semicircular canal, angle, orientation

## Abstract

**Objectives:** To develop and evaluate a new coordinate system for MRI of the vestibular system.

**Methods:** In this study, 53 internal auditory canal MRI and 78 temporal bone CT datasets were analyzed. Mimics Medical software version 21.0 was used to visualize and three-dimensionally reconstruct the image data. We established a new coordinate system, named W–X, based on the center of the bilateral eyeballs and vertex of the bilateral superior semicircular canals. Using the W–X coordinate system and Reid's coordinate system, we measured the orientations of the planes of the anterior semicircular canal (ASCC), the lateral semicircular canal (LSCC), and the posterior semicircular canal (PSCC).

**Results:** No significant differences between the angles measured using CT and MRI were found for any of the semicircular canal planes (*p* > 0.05). No statistical differences were found between the angles measured using Reid's coordinate system (CT) and the W–X coordinate system (MRI). The mean values of ∠ASCC & LSCC, ∠ASCC & PSCC, and ∠LSCC & PSCC were 84.67 ± 5.76, 94.21 ± 3.81, and 91.79 ± 5.22 degrees, respectively. The angle between the LSCC plane and the horizontal imaging plane was 15.64 ± 3.92 degrees, and the angle between the PSCC plane and the sagittal imaging plane was 48.79 ± 4.46 degrees.

**Conclusion:** A new W–X coordinate system was developed for MRI studies of the vestibular system and can be used to measure the orientations of the semicircular canals.

## Introduction

The annual prevalence of dizziness is approximately 11% in the United States (1, 2) and dysfunction of the vestibular system is one of the most common causes. The vestibular apparatus is very small and located on the deep side of the temporal bone (3). Two specific structures of the vestibular system, the otolith organs and semicircular canals, enable humans to perceive head rotation, angular acceleration, and spatial orientation (4). The orientations of the semicircular canals affect the sensitivity of the vestibule to angular acceleration (5, 6), and animals with more perpendicular canals encounter higher head angular velocity during locomotion (7). Thus, an accurate knowledge of the orientations of the semicircular canals is critical to understanding vestibular function and disease (8). The direction of the semicircular canals may determine the dynamics of endolymph flow, which in turn affects the sensory hair cells and ultimately the sensitivity of the tube system. Approximately 10–20% of patients with suspected benign paroxysmal positional vertigo (BPPV) cannot be accurately diagnosed or effectively treated using routine procedures (9, 10), which involve a series of head rotations at specific angles. For these subjects, the relative orientations of the semicircular canals may play a critical role in diagnostic complications and the effectiveness of treatment maneuvers (11).

Measuring the orientations of the semicircular canals requires a reliable three-dimensional (3D) coordinate system for the human brain. Frankfurt and Reid coordinate systems are commonly used in CT (12, 13), but these rely on bony landmarks that are difficult to identify accurately using MRI. Moreover, CT exposes subjects to radiation and cannot resolve the membranous labyrinth (14). Suzuki et al. used the total foot bifurcation point of the semicircular canals and the eyeball midpoint to determine the horizontal plane (HP) (15). Aoki et al. found that Reid's horizontal canal plane lies very close to the HP defined by Suzuki (16). However, different orientations of the semicircular canals have been reported using different coordinate systems (11, 17, 18). In this study, we introduced and assessed a new coordinate system for MRI of the vestibular system.

## Materials and Methods

### Patients and Data Acquisition

Imaging data were obtained from patients who underwent temporal bone high-resolution CT (Revolution 256-slice CT, GE Healthcare, IL, USA) or internal auditory canal MRI (Signa HDx 3.0T, GE Healthcare, IL, USA; Ingenia 3.0T, Philips, Netherlands) examinations at The First Affiliated Hospital of Guangzhou Medical University between December 1, 2018 and March 1, 2021. The scanning procedures followed standard temporal bone CT and inner ear MRI imaging protocols. CT (120 kV, 250 mA, FOV: 320 mm, matrix: 512 ×512, and slice thickness: 0.625 mm), 3D fast-imaging using steady-state acquisition (FIESTA) MRI (GE, TR: 6.02 ms, TE: 2.85 ms, FOV: 180 mm, matrix: 512 ×512, and slice thickness: 0.50 mm), and T2W-3D-DRIVE MRI (Philips, TR: 2000 ms, TE: 200 ms, FOV: 150 mm, matrix: 480 ×480, and slice thickness: 0.50 mm) were performed independently. Inclusion criteria were the clear visualization of the anatomical structures of the cochlea and semicircular canals. The exclusion criterion was an inner ear deformity or superior semicircular canal dehiscence. The final dataset included 123 patients, 78 CT scans (male: 37, female: 41), and 53 MRI scans (GE, male: 35, female: 12; Philips, male: 2, female: 6). Both CT and MRI data were available for 8 patients. The study protocol was reviewed and approved by The First Affiliated Hospital of Guangzhou Medical University review board, Guangzhou, Guangdong, China.

### Image Analysis and Coordinate Systems

Digital Imaging and Communication in Medicine (DICOM) image files for CT and MRI data contain positioning data that define the spatial coordinate in the super–inferior, anteroposterior, and left–right directions. Mimics Medical software version 21.0 was used to view these images and reconstruct these images in 3D images. We used Reid's coordinate system for CT images (12) and a new W–X coordinate system for MR images.

**Figure 1:**
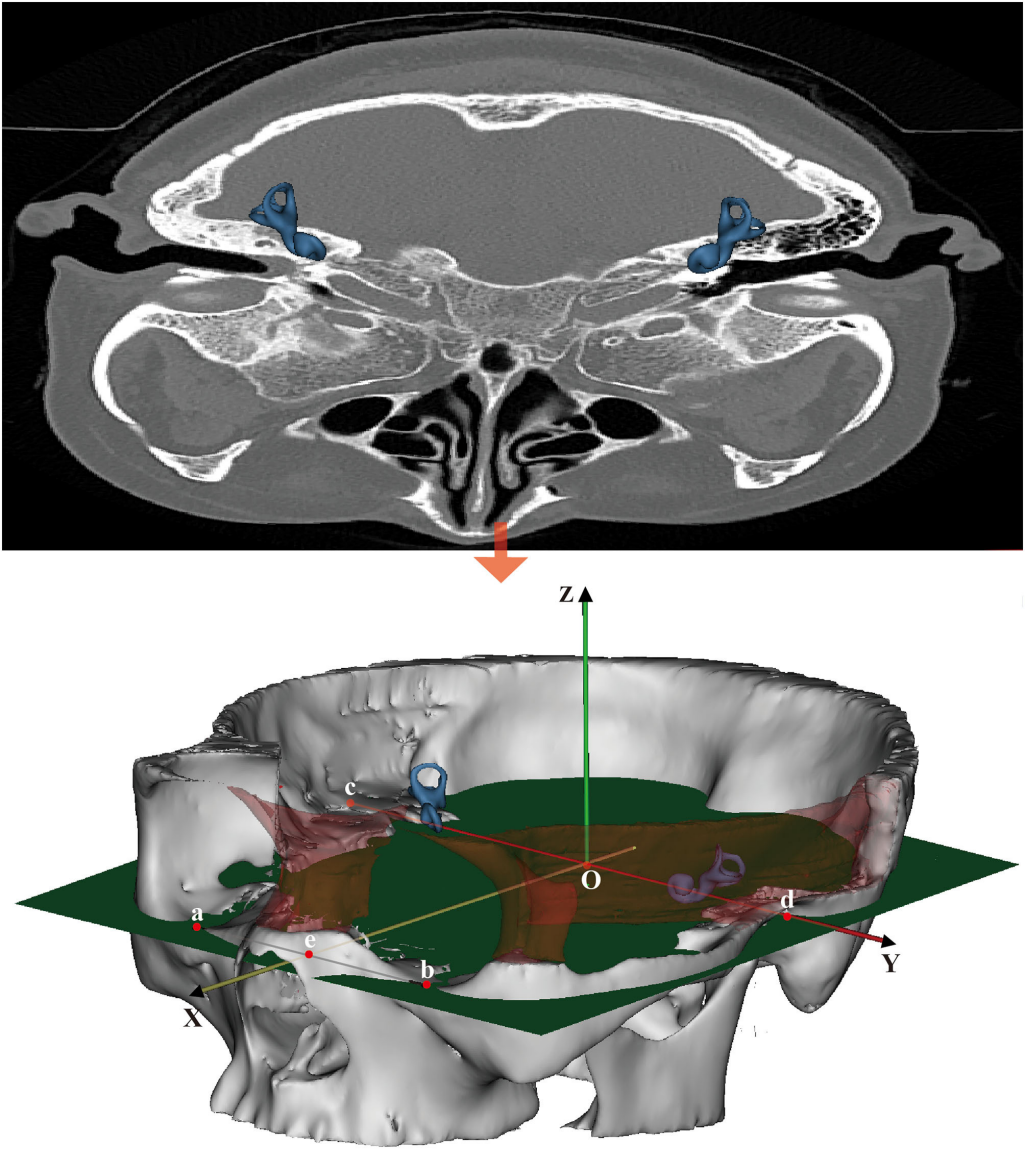
Reid's coordinate system overlaid on a CT image. Points a and b are the bilateral midpoints of the inferior margin of the orbits. Points c and d are the bilateral midpoints of the external auditory canal. Point e is the midpoint of the line segment $\bar{\text{ab}}$. The origin of the coordinate system, point O, is the midpoint of line segment $\bar{\text{cd}}$. The *x-* and *y-*axes are defined by the rays $\vec{\text{Oe}}$ and $\vec{\text{Od}}$, respectively, and the *z*-axis is perpendicular to *x* and *y* (+superior). The sagittal plane (SP) was defined as the plane that contains point O and is perpendicular to the *y*-axis.

Reid's coordinate system is based on anatomical landmarks independent of the head position of the subject during the CT examination. Reid's horizontal plane (R-HP) is defined to pass through the inferior margin of the two orbits and bilateral midpoint of external auditory canals. Reid's sagittal plane (R-SP) is defined as the plane that lies perpendicular to R-HP and connects the left–right orbital midpoint and the bilateral midpoint of the opening of external auditory canals. Reid's frontal plane (R-FP) is defined as the plane that lies perpendicular to both R-HP and R-SP and passes through the midpoint of the line connecting the tips of the bilateral midpoint of the external auditory canals (Figure 1).

**Figure 2:**
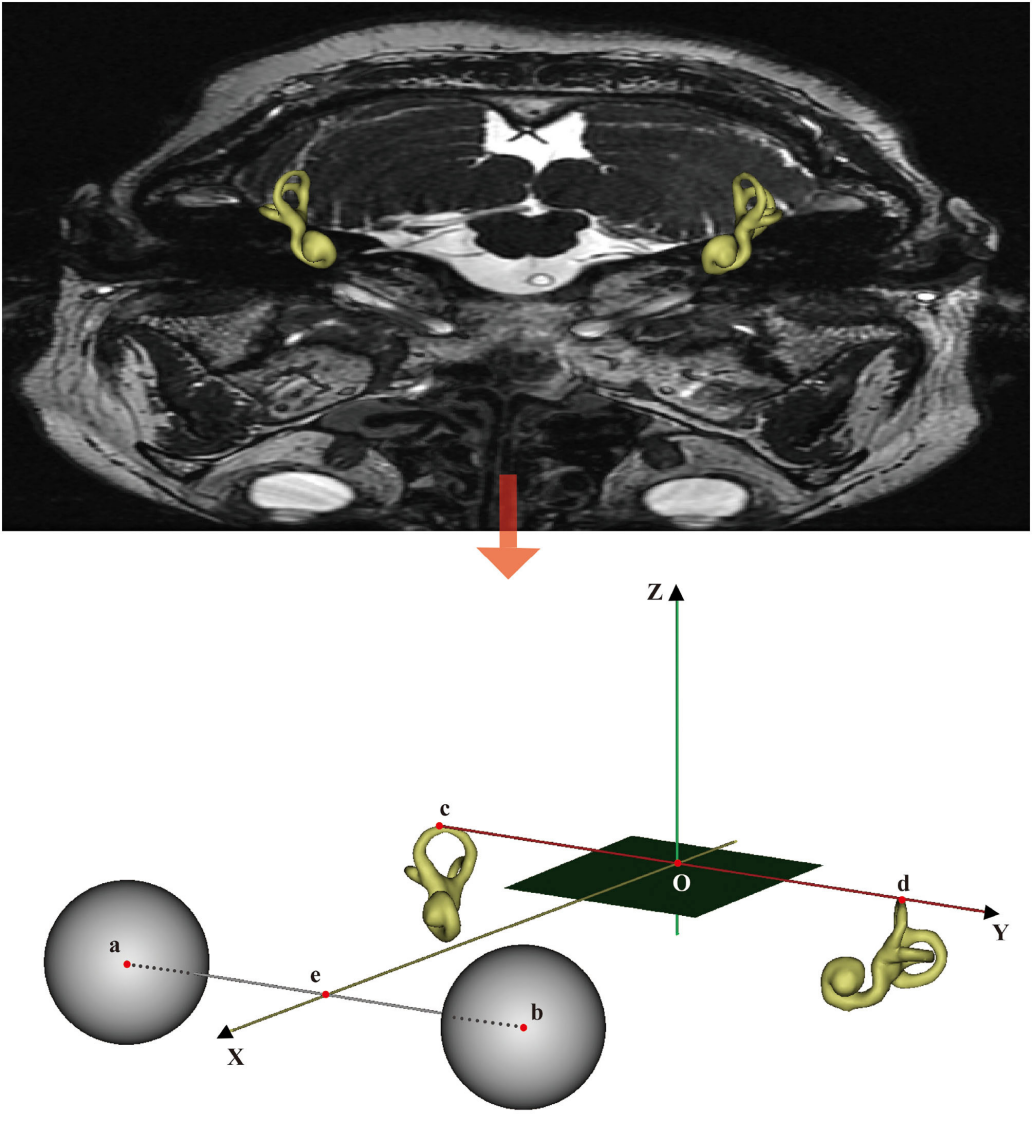
The W–X coordinate system overlaid on a CT image. Points a and b are the centers of the two eyeballs. Points c and d are the vertices of the bilateral superior semicircular canals. Point e is the midpoint of the line segment $\bar{\text{ab}}$. The origin of the coordinate system, point O, is the midpoint of line segment $\bar{\text{cd}}$. The *x-* and *y*-axes are defined by the rays $\vec{\text{Oe}}$ and $\vec{\text{Od}}$, respectively, and the *z*-axis is normal to the horizontal plane containing *x* and *y* (+superior). The SP is defined as the plane that contains point O and is perpendicular to the *y*-axis.

The W–X horizontal plane (W-HP) was defined as the plane passing through the center of the bilateral eyeballs and vertex of the bilateral superior semicircular canals. The W–X sagittal plane (W-SP) was defined as the plane that lies perpendicular to W-HP and connects the center of the bilateral eyeball midpoint and the line connecting the tips of the vertex of the bilateral superior semicircular canals. The W–X frontal plane (W-FP) was defined as the plane that lies perpendicular to both W-HP and W-SP and passes through the midpoint of the line connecting the tips of the bilateral midpoint of external auditory canals (Figure 2).

**Figure 3:**
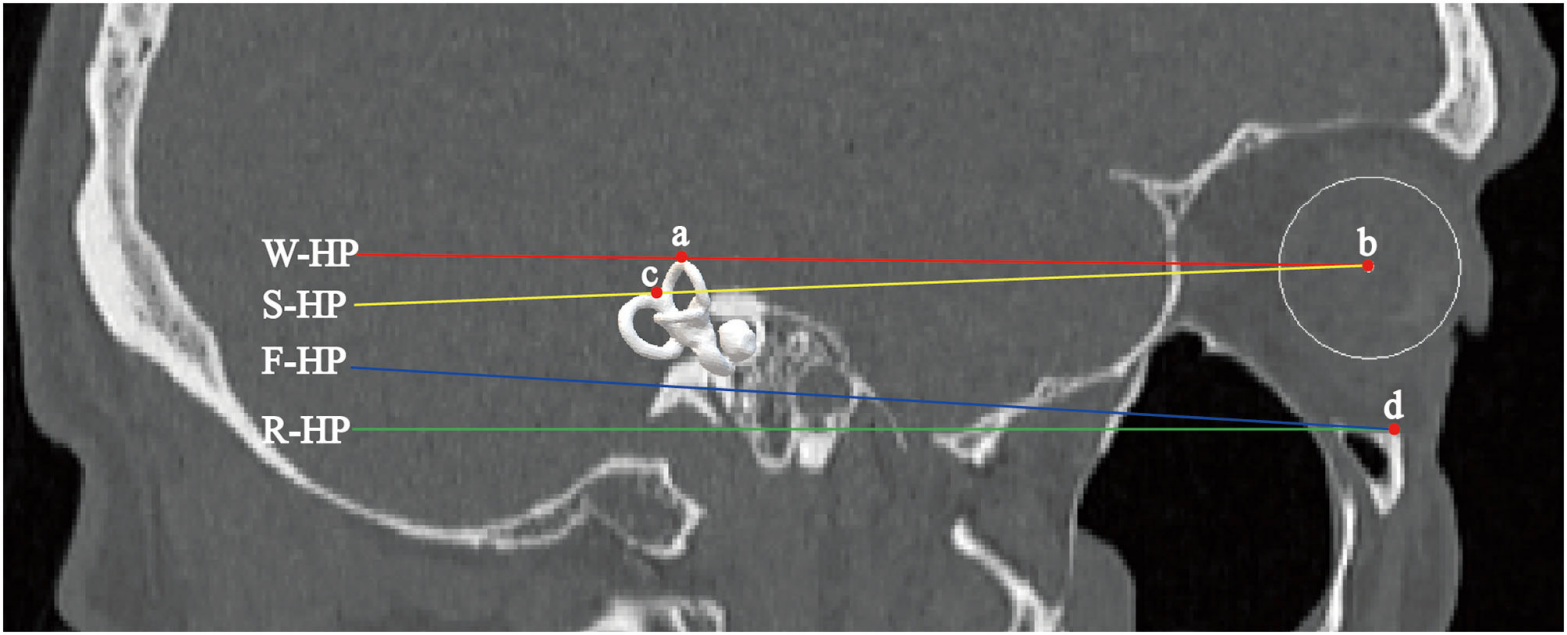
Four different coordinate planes. Point a is the vertex of the bilateral superior semicircular canals. Point b is the center of the eyeball. Point c is the bifurcation of the common crus. Point d is the inferior margin of the orbit. W-HP, S-HP, F-HP, and R-HP refer to the horizontal planes of the W–X, Suzuki, Frankfurt, and Reid coordinate systems, respectively.

In addition, we established and compared differences among the R-HP, W-HP, Frankfurt horizontal plane (F-HP), and Suzuki horizontal plane (S-HP) (Figure 3). F-HP is the plane passing through the bilateral vertex of the opening of the external auditory meatus and the left infraorbital edge point (17). S-HP was defined as the plane passing through the center of the bilateral eyeballs and the bilateral bifurcations of the common crus (15).

### Angle Measurement

**Figure 4:**
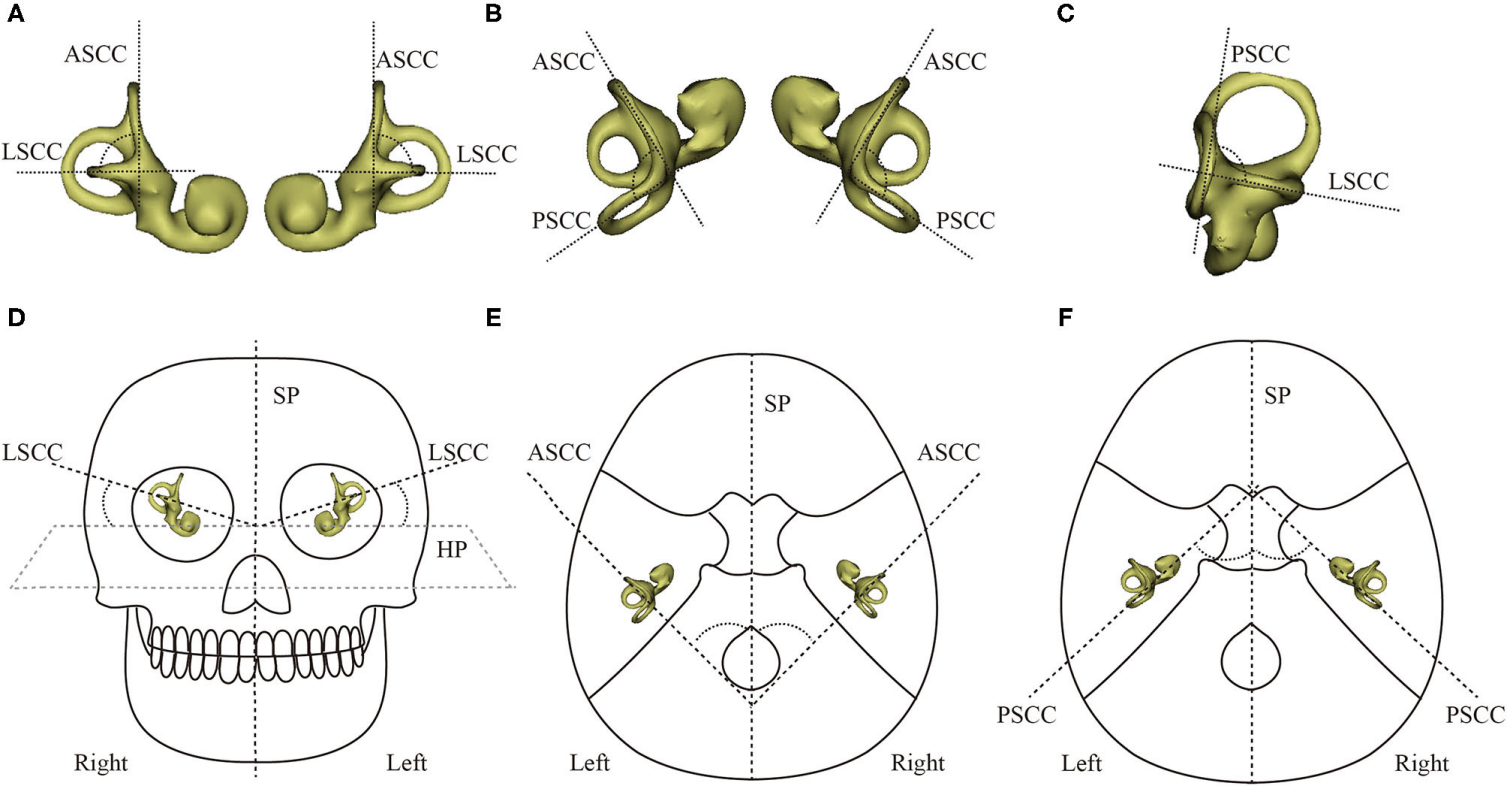
The angles between the planes of the semicircular canals and between the canal planes and the imaging planes. **(A)** ASCC & LSCC. **(B)** ASCC & PSCC. **(C)** PSCC & LSCC. **(D)** ∠LSCC & HP. **(E)** ∠ASCC & SP. **(F)** ∠LSCC & SP. ASCC, PSCC, and LSCC refer to the anterior, posterior, and lateral semicircular canals, respectively; SP, HP, and FP, respectively, refer to the sagittal, horizontal, and frontal imaging planes.

The relative orientations of semicircular canals within the cranial base were defined as the angles between the standard sagittal plane (SP), horizontal plane (HP), and frontal plane (FP) and each canal plane (Figure 4). The anterior, posterior, and lateral semicircular canals were referred to as ASCC, PSCC, and LSCC, respectively. The “fit center line” tool of the Mimics Medical software was used to obtain the centerline and 3D coordinate (*x, y, z*) of the center point of each semicircular canal. A multipoint least-squares fitting method was used to define the canal planes (20–40 center positions in each section) and four points of the W-HP (12). Coordinate values were imported into an in-house program written in the MATLAB (MathWorks Inc., Natick, MA, USA) programming language. Two investigators measured the relative orientations of the semicircular canals independently.

### Statistical Analysis

Statistical analysis was performed using SPSS version 25.0 statistical software (SPSS Inc, IBM, Chicago, IL, USA). Data with normal or approximately normal distributions are represented as mean ± SD. Differences between groups were measured using a *t*-test. All quoted *p* values are two-sided and *p* < 0.05 was considered statistically significant.

## Results

### Angular Relationships Between Semicircular Canals

**Figure 5:**
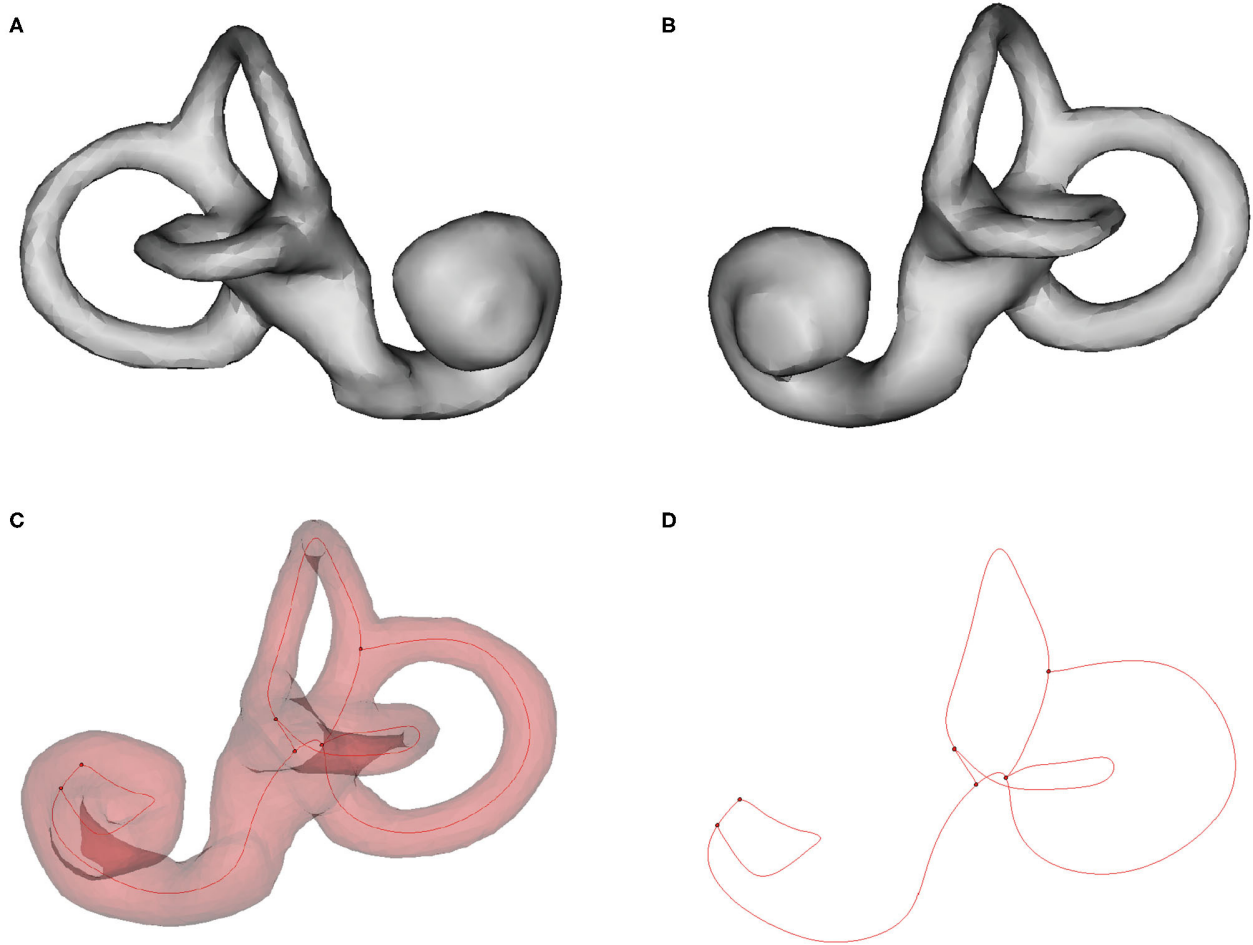
Three-dimensional rendering of the cochlea and semicircular canals, and the centerline of the semicircular canals. **(A)** Left cochlea and semicircular canal. **(B)** Right cochlea and semicircular canal. **(C, D)** Centerline (red) of the right semicircular canal.

The 3D model of the cochlea and semicircular canals is shown in Figures 5A,B. The centerline of the semicircular canals shows their morphological characteristics (Figures 5C,D). We measured the relative orientations of the semicircular canal planes (Table 1). There were no statistically significant differences between the angles measured using CT and MRI for any of the ipsilateral or contralateral semicircular canal planes. Therefore, the relative angles between the planes of semicircular canals measured using CT and MRI can be combined, yielding mean values for ∠ASCC & LSCC, ∠ASCC & PSCC, and ∠LSCC & PSCC of 84.67 ± 5.76, 94.21 ± 3.81, and 91.79 ± 5.22 degrees, respectively.

**Table 1:** Angles (degrees) between the planes of the semicircular canals, measured using CT and MRI.

**Angle**	**CT (*n* = 78)**		**MRI (*n* = 53)**			
	**Mean**	***SD***	**Mean**	***SD***	**t**	***p***
∠ASCC& LSCC	84.2176	6.05167	85.3321	5.29825	−1.087	0.279
∠ASCC & PSCC	94.7569	3.81805	93.4272	3.69325	1.982	0.050
∠LSCC & PSCC	92.0028	5.89758	91.4793	4.06055	0.602	0.548
∠R-ASCC & L-ASCC	75.095	7.58761	75.4098	6.85802	−0.242	0.809
∠L-LSCC & R-LSCC	167.9807	7.27765	165.9583	8.49	1.459	0.147
∠R-PSCC & L-PSCC	95.2038	7.59068	97.7854	6.97694	−1.973	0.051
∠L-ASCC & R-PSCC	15.3473	5.73015	15.9285	4.85228	−0.605	0.546
∠R-ASCC & L-PSCC	16.0313	5.45998	16.2188	4.51145	−0.207	0.837

*ASCC, anterior semicircular canal; PSCC, posterior semicircular canal; LSCC, lateral semicircular canal. Prefixes “L-” and “R-” refer to canals on the left and right, respectively*.

### Angles Between the LSCC and Four Different Coordinate Planes

The angles between each of the horizontal reference planes and between each reference plane and the LSCC plane are shown in Table 2. The angle between the R-HP and W–X plane was relatively small. There were no statistically significant differences between the values of ∠LSCC & W-HP and ∠LSCC & R-HP (*t* = 1.62, *p* = 0.11). However, there were statistical differences between ∠LSCC & R-HP and ∠LSCC & F-HP (*t* = 7.7, *p* < 0.01), and between ∠LSCC & S-HP and ∠LSCC & R-HP (*t* = −15.86, *p* < 0.01). Therefore, W-HP can be used in place of R-HP to establish the horizontal plane for MRI.

**Table 2:** Angles (degrees) between the LSCC plane and the horizontal planes of the W–X horizontal plane.

**Angles**	**Mean**	**SD**
∠W-HP & R-HP	1.95	1.01
∠S-HP & R-HP	5.18	1.42
∠F-HP & R-HP	4.70	1.22
∠W-HP & F-HP	6.39	1.74
∠S-HP & F-HP	9.62	1.93
∠LSCC & W-HP	15.23	4.56
∠LSCC & R-HP	15.63	4.08
∠LSCC & S-HP	11.7	5.61
∠LSCC & F-HP	18.2	4.74

*(W-HP), Reid horizontal plane (R-HP), Frankfurt horizontal plane (F-HP), and Suzuki horizontal plane (S-HP) coordinate systems*.

### Orientations of Semicircular Canals

No statistically significant differences were observed between the orientations of the semicircular canals measured using Reid's coordinate system in CT and the W–X coordinate system in MRI (Table 3). Therefore, the angles measured using CT and MRI can be combined. The angles between the ASCC plane and W-SP, W-HP, and W-FP were thus 39.21 ± 4.22, 112.39 ± 6.8, and 124.60 ± 3.69 degrees, respectively; the angles between the LSCC plane and SP, HP, and FP were 91.08 ± 5.01, 15.64 ± 3.92, and 102.83 ± 5.44 degrees, respectively; and the respective angles between the PSCC plane and SP, HP, and FP were 48.79 ± 4.46, 77.38 ± 6.28, and 134.53 ± 3.80 degrees. To confirm that the W–X coordinate system can replace Reid's system, we analyzed data from patients who underwent both CT (Reid's system) and MRI (W–X system) and found no statistical differences between the angles measured using either system (Table 4).

**Table 3:** Angles (degrees) between the planes of the semicircular canals and the sagittal plane (SP), horizontal plane (HP), and frontal plane (FP).

**Angle**	**CT (*n* = 78)**		**MRI (*n* = 53)**			
	**Mean**	**SD**	**Mean**	**SD**	**t**	***p***
∠ASCC & SP	39.36	4.48	38.98	3.84	0.50	0.62
∠ASCC & HP	113.10	6.63	111.34	6.98	1.46	0.15
∠ASCC & FP	124.62	4.03	124.58	3.18	0.06	0.95
∠LSCC & SP	90.76	4.84	91.54	5.28	−0.87	0.38
∠LSCC & HP	15.59	3.47	15.72	4.53	−0.19	0.85
∠LSCC & FP	102.49	5.52	103.35	5.34	−0.88	0.38
∠PSCC & SP	48.42	4.71	49.34	4.04	−1.17	0.24
∠PSCC & HP	77.64	6.38	77.01	6.18	0.56	0.58
∠PSCC & FP	134.11	3.94	135.15	3.53	−1.53	0.13

**Table 4:** Angles (degrees) measured in the same patient between the planes of the semicircular canals and between the canal planes and the imaging planes.

**Angle**	**CT (*n* = 8)**		**MRI (*n* = 8)**			
	**Mean**	**SD**	**Mean**	**SD**	**t**	***p***
∠ASCC& LSCC	86.93	2.94	85.68	5.62	0.55	0.60
∠ASCC & PSCC	94.86	2.66	93.22	4.25	2.20	0.06
∠LSCC & PSCC	90.24	4.67	91.44	3.58	−1.33	0.23
∠R-ASCC & L-ASCC	72.20	9.60	73.96	9.32	−1.73	0.13
∠L-LSCC & R-LSCC	168.48	7.12	166.61	7.13	0.59	0.57
∠R-PSCC & L-PSCC	99.31	8.70	101.73	6.41	−1.93	0.10
∠L-ASCC & R-PSCC	17.24	7.60	19.30	6.44	−1.37	0.21
∠R-ASCC & L-PSCC	16.61	8.61	16.59	6.13	0.01	0.99
∠ASCC & SP	36.31	4.97	37.11	4.68	−1.65	0.14
∠ASCC & HP	98.89	5.82	98.38	6.84	0.39	0.71
∠ASCC & FP	124.17	3.43	124.97	2.14	−1.11	0.30
∠LSCC & SP	90.83	4.30	89.98	4.06	0.43	0.68
∠LSCC & HP	15.20	3.12	14.41	3.81	0.98	0.36
∠LSCC & FP	103.37	3.28	102.18	4.77	0.65	0.54
∠PSCC & SP	49.86	4.30	51.02	3.17	−1.90	0.10
∠PSCC & HP	78.24	6.94	76.89	7.08	2.27	0.06
∠PSCC & FP	136.58	3.99	137.01	2.48	−0.64	0.54

## Discussion

We introduced a new W–X coordinate system for MRI that uses the eyeballs and anterior semicircular canals as reference points. It is challenging to accurately fix the head position during CT and MRI examinations, so coordinate data in the file header of the DICOM image file may contain positioning errors. The W–X coordinate system can solve this problem for stereotactic MRI analyses of cranial organs. Previous studies have commonly used Frankfurt and Reid coordinate systems, whose alignments usually rely on bony markers, such as the external auditory meatus and infraorbital ridge (12). However, it is difficult to accurately identify bony markers using MRI because air and bone yield low signal intensity. Suzuki et al. proposed an MRI coordinate system that uses the total foot bifurcation point of the semicircular canal and the eyeball midpoint to define the horizontal plane, S-HP (15). Aoki et al. found that R-HP lies very close to the S-HP (16). Conversely, we found a statistical difference between ∠LSCC & S-HP and ∠LSCC & R-HP. In addition, no statistical differences were found between the angles of semicircular canals measured using Reid's coordinate system (CT) and the W–X coordinate system (MRI). Therefore, the W–X coordinate system can be used to analyze both CT and MRI data. In addition, all inner ear structures can be accurately mapped in the W–X coordinate system because this system is uniquely defined for each subject. Additionally, the W–X coordinate system can compensate for the structural complexity and measurement uncertainty of the midpoint of the lower edge of the orbit and the center of the external auditory canal in Reid's coordinate system.

MRI is more suitable than CT for routine anatomical research because it avoids exposure of subjects to radiation. In addition, it can visualize in detail the membrane labyrinth, peripheral nerve, and blood vessel structures from multiple angles and quantify the structure and volume of the inner ear (19). CT has long been used to assess the morphology of the temporal bone and assess the extent of lesions in the ossicular chain and bone labyrinth, but it cannot detect the membrane labyrinth (14). Membrane semicircular canals were reported to deviate from the bone semicircular canals by 2–6 degrees (13). However, our results showed no significant differences between CT and MRI images of these structures. The reason for this may be that the bone labyrinth is filled with internal and perilymph fluids but MRI mainly detects anatomical structures containing fluid (20).

The W–X coordinate system provides a new means for measuring the orientations of the semicircular canals. Measuring the relative orientations and positions of the semicircular canals can be used for preoperative planning of surgical procedures, such as cochlear implantation (21, 22). It can provide advance knowledge of the location and degree of inner ear deformity and changes in labyrinthine shape, which can be used to develop personalized treatment plans. In addition, *in vitro* modeling of the semicircular canal requires precise measurement of the relative orientations and positions of the semicircular canals (23).

We found that the planes of adjacent semicircular canals were not perpendicular and the bilateral horizontal semicircular canals were not coplanar. To measure relative orientations of the semicircular canals, the plane of each semicircular canal needs to be obtained. However, the semicircular canals are not strictly planar, and the anterior canal showed the largest torsion compared with the others (24, 25). There exist no uniform criteria to best identify the approximate planes of the semicircular canals, and different methods may yield different results. Using the three points at the center of the cross-section of the canals (the ampulla and non-ampullated end, bifurcation at the vertical canals, and their approximate midpoint) to define a semicircular canal is the most common method, which can be easily performed in minimal time (15–18, 24, 26, 27). However, it cannot reflect the curvature of the semicircular canal and results are difficult to reproduce because of the inherent subjectivity. Direct measurement of the angle between the coordinate plane and the maximum cross-section of the semicircular canal (11, 28, 29) is simple but too subjective. Among them, the most objective approach is a multipoint fitting method, which defines the canal planes by calculation of 20–40 center positions in each cross-section of the canals using a least-squares method (12, 13, 30) and can accurately describe the morphological structure of semicircular canals (25). In this study, we used third-party software to obtain the centerline and center points of the semicircular canals, reducing the error associated with manual point-picking. These center points were then used for the multipoint calculation of the canal planes. Therefore, the angles measured in this study are considered reliable.

Accurate structural information about the semicircular canals can aid the clinical diagnosis and treatment. Excluding patients with an inner ear deformity or superior semicircular canal dehiscence, we can use the orientations of semicircular canals to improve or develop a new BPPV treatment to reposition the otolith (31, 32). Approximately 10–20% of patients with suspected BPPV cannot be accurately diagnosed or effectively treated using routine procedures, such as the Dix–Hallpike, Epley, or Semont maneuvers (9, 10), which may be due to individual differences in the orientations of the semicircular canals and the speed of operation (11, 12, 33). The Dix–Hallpike maneuver is performed by moving the patient from an upright to a supine position, with the head rotated by 45 degrees to one side, the neck extended by 20 degrees, and the affected ear facing down (31). We found that the average angle between the plane of the PSCC and the SP was 48.79 degrees, and the average angle between the LSCC and the HP was 15.64 degrees. This means that bowing the head by approximately 49 degrees and then rotating it to the right (left) by ~16 degrees will result in the coplanar alignment of the right (left) PSCC and the SP. Knowledge of the canal geometry is important for accurate stimulation of the LSCC during the roll test. This test assumes that the angle between the LSCC and the HP is 30 degrees, but our results indicate that this angle is 15.64 degrees. The latter angle is more conducive for moving the otolith from the PSCC into the vestibule. These findings suggest that accurate measurement of the semicircular canal geometry can be used to develop personalized BPPV treatment plans, especially for patients who have not responded to the repositioning therapy.

In summary, a new W–X coordinate system was developed for MRI studies of the vestibular system and can reliably measure the orientations of the semicircular canals.

## Data Availability Statement

The raw data supporting the conclusions of this article will be made available by the authors, without undue reservation.

## Ethics Statement

The studies involving human participants were reviewed and approved by the First Affiliated Hospital of Guangzhou Medical University Institutional Review Board. Written informed consent from the patients/ participants or patients/participants' legal guardian/next of kin was not required to participate in this study in accordance with the national legislation and the institutional requirements.

## Author Contributions

WL, XZ, and LS: conception and design. XZ: administrative support. WL, GC, JX, and TL: provision of study materials or patient data. WL, GC, JX, TL, CZ, XL, WL, and LS: collection and assembly of data. WL, GC, JX, TL, and LS: data analysis and interpretation. All the authors have written and approved the final manuscript.

## Funding

This work was supported by the Science and Technology Planning Project of Guangdong Province (grant 2016A020220019), the Upper Respiratory Disease Innovation and Transformation Platform Construction Project of Guangdong Provincial, and the High-level Construction Project of Guangzhou Medical University.

## Conflict of Interest

The authors declare that the research was conducted in the absence of any commercial or financial relationships that could be construed as a potential conflict of interest.

## Publisher's Note

All claims expressed in this article are solely those of the authors and do not necessarily represent those of their affiliated organizations, or those of the publisher, the editors and the reviewers. Any product that may be evaluated in this article, or claim that may be made by its manufacturer, is not guaranteed or endorsed by the publisher.

## Abbreviations

ASCC: anterior semicircular canal
PSCC: posterior semicircular canal
LSCC: lateral semicircular canal
HP: horizontal plane
SP: sagittal plane
FP: frontal plane
W-HP: W–X horizontal plane
R-HP: Reid's horizontal plane
F-HP: Frankfurt horizontal plane
S-HP: Suzuki's horizontal plane.
